# Temperature and food quantity effects on the harpacticoid copepod *Nitocra spinipes*: Combining *in vivo* bioassays with population modeling

**DOI:** 10.1371/journal.pone.0174384

**Published:** 2017-03-23

**Authors:** Josef Koch, Thuy T. Bui, Elin Lundström Belleza, Markus Brinkmann, Henner Hollert, Magnus Breitholtz

**Affiliations:** 1 Laboratory of Environmental Toxicology and Aquatic Ecology, Environmental Toxicology Unit (GhEnToxLab), Ghent University, Ghent, Belgium; 2 Department of Environmental Science and Analytical Chemistry, Stockholm University, Stockholm, Sweden; 3 IVL Swedish Environmental Research Institute, Stockholm, Sweden; 4 Toxicology Centre, University of Saskatchewan, Saskatoon, Canada; 5 Institute for Environmental Research, RWTH Aachen University, Aachen, Germany; Charles University, CZECH REPUBLIC

## Abstract

The harpacticoid copepod *Nitocra spinipes* has become a popular model species for toxicity testing over the past few decades. However, the combined influence of temperature and food shortage, two climate change-related stressors, has never been assessed in this species. Consequently, effects of three temperatures (15, 20 and 25°C) and six food regimes (between 0 and 5 × 10^5^ algal cells/mL) on the life cycle of *N*. *spinipes* were examined in this study. Similarly to other copepod species, development times and brood sizes decreased with rising temperatures. Mortality was lowest in the 20°C temperature setup, indicating a close-by temperature optimum for this species. Decreasing food concentrations led to increased development times, higher mortality and a reduction in brood size. A sex ratio shift toward more females per male was observed for increasing temperatures, while no significant relationship with food concentration was found. Temperature and food functions for each endpoint were integrated into an existing individual-based population model for *N*. *spinipes* which in the future may serve as an extrapolation tool in environmental risk assessment. The model was able to accurately reproduce the experimental data in subsequent verification simulations. We suggest that temperature, food shortage, and potentially other climate change-related stressors should be considered in environmental risk assessment of chemicals to account for non-optimal exposure conditions that may occur in the field. Furthermore, we advocate combining *in vivo* bioassays with population modeling as a cost effective higher tier approach to assess such considerations.

## Introduction

Large amounts of man-made and naturally occurring substances continuously enter the environment, where they may ultimately cause adverse effects in living organisms. Information about the potential toxicity of chemicals in the aquatic environment is commonly derived from toxicity tests using only a few aquatic species, chosen to be representative of different trophic levels in the food web. While parthenogenic cladocerans of the genus *Daphnia* are widely used as model organisms to evaluate the hazardous potential of chemicals toward primary consumers in their entirety [1, 2], the sexually reproducing Copepoda form another group of valuable test species. Insights into copepod reproduction dynamics in stressful environments can be helpful for understanding population-level effects of sexually reproducing crustaceans in general. Furthermore, in contrast to daphnids who can solely be found in limnic environments, copepods also play a key role in the world’s oceans [3]. Harpacticoida, for instance, forge an important trophic link between the microphytobenthos and higher trophic levels in marine and brackish environments [3–5]. The euryhaline brackish water copepod *Nitocra spinipes* was introduced as an aquatic testing organism in ecotoxicological studies in the 1970s [6] and has since then been used extensively for the evaluation of a wide range of chemicals, effluents, sediments etc. [7–9]. In 2014, the Organisation for Economic Co-operation and Development (OECD) released a guidance document on the harpacticoid copepod development and reproduction test with *Amphiascus tenuiremis* [10]. The validation work behind this test initially included *N*. *spinipes* but a decision was taken to proceed with *A*. *tenuiremis* as the sole species due to slightly better performance [11, 12]. However, it has been shown that the test can be successfully applied also with *N*. *spinipes* [13, 14]. *N*. *spinipes* undergoes multiple molts during its maturation process. Newly hatched animals pass through six naupliar and five copepodite stages before reaching adulthood. After mating, females can produce up to 6 or more broods with sometimes over 40 nauplii per brood [15].

A considerable amount of research has been conducted to assess the effects of different compounds on the development, reproduction and mortality of *N*. *spinipes* and other harpacticoid species at favorable laboratory conditions [e.g. 16–18] However, only a few experiments aimed to assess the influence of different exposure temperatures and food quantities on individual organism traits [19, 20]. According to Huntley and Lopez [21], 90% of the variance in separately published estimates of reproduction time for 33 copepod species could be explained by the temperature alone.

Global climate change has brought significant shifts in the timing of seasonal temperatures. Spring temperatures in the oceans of the Northern Hemisphere have occurred earlier by two days per decade since 1960 [22]. Different responses to this phenomenon cause a trophic mismatch within the ecological community [23, 24] which in turn leads to periods of food shortage for marine copepods and other species [25]. Furthermore, climate change has led to changes in the proportion of dinoflagellates and diatoms in the northeast Atlantic, the North Sea and the Baltic Sea [26, 27] which may have a direct negative effect on copepods and other grazers, some of which are selective with respect to their food source [28]. Since suboptimal conditions of temperature, food, and other natural stressors can increase a species’ sensitivity toward chemical stress on the individual and population level [29–32], it has earlier been proposed that climate change-induced variations in these factors should be considered in environmental risk assessment of chemicals [33, 34].

Finding accurate temperature functions based on physiological knowledge has been a great challenge in ecological research. Since all metabolic and physiological processes are based on chemical reactions, various authors applied the Arrhenius equation to describe development rates of ectotherms [35–37]. However, many data sets showed curvilinear relationships, not satisfactorily covered by Arrhenius’s theoretical expressions [38]. Despite the lack of a fully mechanistic basis, Bělehrádek’s function has become one of the most popular functions to describe the temperature dependency of copepod development times [20, 38–40].

Decreasing food quantities have generally been found to prolong copepod development times [41–43] and increase mortality [41, 44]. In contrast to temperature, however, the link between food availability and copepod life cycle processes is less direct. Especially under semi-static feeding conditions, fluctuations in the food concentration occur due to degradation and consumption, affecting the copepods’ ingestion rate.

The aims of this study were (a) to analyze and quantify the effects of temperature and (b) food availability on the development, reproduction, sex ratio and mortality of the harpacticoid copepod *N*. *spinipes* as a standard test species that has been used extensively for toxicity testing over the last decades, and (c) to integrate the corresponding mathematical functions into an existing individual-based population model for this species [14].

## Material and methods

### Test species

The strain of *N*. *spinipes* used in the present study was isolated by ourselves from sediments in the Tvären Bay, Baltic Sea in 1975 and has been in continuous culture ever since [45]. The culture is permanently maintained in darkness at 22 ± 1°C. Detailed information on the laboratory culture of this strain has been published elsewhere [45, 46].

### Life cycle experiments

Life cycle experiments were performed to assess the effects of different temperatures and food concentrations on individual level traits of *N*. *spinipes*. The experimental setups were chosen in accordance with methods tested and proven by Lundström Belleza [15]. These methods consider the same endpoints as the harpacticoid copepod development and reproduction test with *A*. *tenuiremis* [10] but were optimized for better manageability with *N*. *spinipes*. Microplate wells of different volumes were used as incubation vessels. Brackish sea water, which had been filtered with glass microfiber filters (30 μm), heated to 80°C and recooled to room temperature, respectively, was used as cultivation medium. Animals were fed with the cryptophytic alga *Rhodomonas salina*. This species was selected as it has proven to be a suitable food item for copepods [47] and *N*. *spinipes* in particular [48]. For all treatments, the food was replaced every first, third and fifth day of a week. All setups were permanently maintained in darkness except for countings and food replacements.

### Treatments

To investigate temperature dependency, three temperature regimes (‘temperature setups’) were used: 15, 20 and 25°C. An adequate number of stock culture vessels were transferred to the 15 and 25°C setups at least 18 days before the start of the tests to ensure sufficient temperature acclimatization. Animals to be used in the 20°C setup were taken directly from the original stock culture (22 ± 1°C). Actual temperatures during the experiment were recorded as daily minima and maxima. The average temperature per treatment over the whole testing period was estimated as the arithmetic mean of all daily minima and maxima. In all three temperature setups, a food concentration of 2.5 × 10^5^ cells/mL was maintained.

Experiments on food dependency (‘food setups’) were performed at 22 ± 1°C, i.e. the temperature used in the cultures. In seven different treatments, food was offered in concentrations of 0, 0.125, 0.25, 0.5, 1.25, 2.5 and 5 × 10^5^ algal cells/mL.

### Development test

For each temperature setup, 72 newly hatched (< 24 h) nauplii were transferred to wells containing 2 mL of medium (24-well plates) in groups of six. As soon as individuals had reached the first copepodite stage, they were isolated in wells of 270 μL in 96-well plates. For each food setup, 35 nauplii were treated accordingly. All animals were observed over a timespan of at least 30 days. The plates were checked daily for animals that had reached the first copepodite stage and adulthood, respectively. The transition from the last naupliar to the first copepodite stage was easily observed microscopically. To determine the day of reaching adulthood, copepodite exoskeletons were collected after each molt. This was done until five exoskeletons had been counted, indicating the final molt to the adult stage. In the case of dead animals, the day of death was registered. The duration from the first naupliar to the first copepodite stage was recorded as naupliar development time. The duration from the first copepodite stage to reaching adulthood was recorded as copepodite development time. Corresponding to Hart [19], the ratio of the mean copepodite development time *Dc* to the mean naupliar development time *Dn* was calculated (*Dc/Dn*). After reaching sexual maturity, the sex of each individual copepod was determined. For this purpose, copepods were euthanized in a 4% formaldehyde solution and inspected under a light microscope. Males were distinguished from females by detection of spermatophores inside their abdomen. In cases of doubt, males could also be identified by their antenna whose first segments are bigger and more pronounced than the others. The antenna of females, in contrast, are more regular-shaped.

### Reproduction test

For each treatment, 144 newly hatched (< 24 h) nauplii were transferred to wells containing 8 mL of medium (6-well plates) in groups of 24 individuals. In this setup, animals were allowed to reproduce freely after reaching sexual maturity. The plates were checked daily for females carrying egg sacs. Such ovigerous females were isolated and transferred into individual 2 mL-wells (24-well plates). The wells were observed for released eggs and hatched nauplii. The number of newly hatched nauplii was counted after each clutch release. Females were observed over a period long enough to ensure the hatching of at least two broods. For the temperature setups, the inter-clutch periods were recorded as a measure of embryonic development time [38]. Jiménez-Melero et al. [49] found embryonic development to be independent of food for the calanoid copepod *Arctodiaptomus salinus*. However, a multi-day latency before the initiation of each new brood (not only the first as is usual) was found at very low food concentrations. Ultimately, inter-clutch periods were not measured for the food setups in this study due to their minor quantitative importance, compared to brood size, for the total reproductional output of a population (see Discussion section). Since in the 25°C temperature treatment unexpectedly few females produced successful broods, an additional experiment was performed in which 45 ovigerous females were taken directly from the acclimatized stock culture and transferred to individual microplate wells. Those females were observed for clutch release and brood size for six consecutive days.

### Statistical evaluation

All statistical tests were executed by means of the ‘stats’ package in R [50]. For all tests, a significance level α of 5% was chosen. The Shapiro-Wilk test of normality and Bartlett’s test of equal variance were applied as pretests to all metric data, including developmental data and brood size data. All metric data groups failed in either one or both pretests. The Kruskal-Wallis test for multiple comparison with Bonferroni correction was applied to compare all nonparametric data groups within an experiment. Fisher's exact test for count data was applied to the categorical endpoints sex ratio (female or male) and stage mortality (alive or dead).

### Model design and parametrization

**Fig 1:**
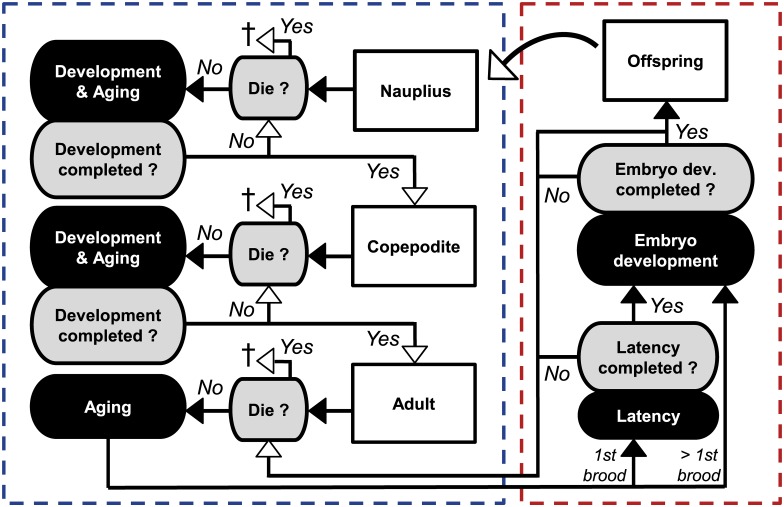
Conceptual diagram of the copepod model. White shaded text boxes represent life stages as state variables. Black shaded text boxes indicate daily development processes, while gray shaded text boxes indicate query for completion of those. Arrows signify the subsequent order of processes within a time step. White arrow tips signify the last process within each particular time step. **†** indicates the death of a copepod. The left box with dashed outlines encloses all processes of development and survival. The right box encloses reproductional processes which are only applied to females.

The individual-based copepod model for *N*. *spinipes* was originally established to optimize the design of the OECD guideline on harpacticoid copepod development and reproduction testing [10, 14]. The individual-based modeling approach was chosen to enable the observation of single individuals over the whole test simulation and to generate realistic variability within the population. The simulated life cycle is shown in Fig 1. Different toxic effect models can be applied to survival and other individual endpoints. A detailed explanation of the model state variables and parameters can be found in the precedent study by Preuss et al. [14].

**Fig 2:**
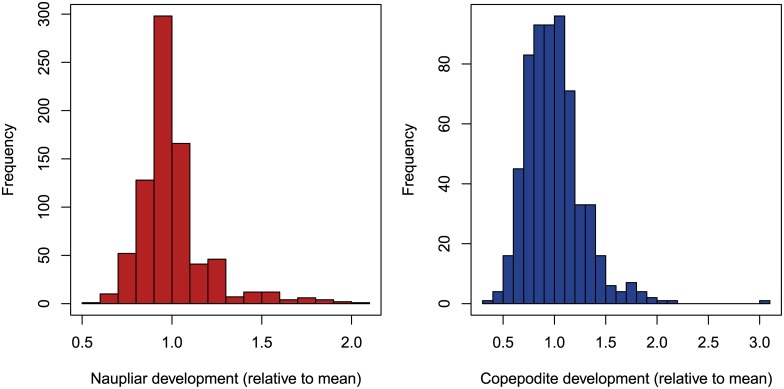
Frequency distributions of naupliar and copepodite development. Frequency distributions of naupliar and copepodite development times are shown for multiple pooled datasets of *N*. *spinipes* which were all normalized to the distribution mean per dataset [11, 13, 15, 48, 51].

All model parameters were initially calculated for optimal conditions of food and temperature. Food was assumed to be available in abundance. The standard temperature was 22°C. Underlying probability distributions of the calculated parameters were also determined and integrated into the model to allow for authentic stochastic variations among individuals. Within the context of this study, the life cycle was reparameterized on the previously used data sets [11, 13, 51] and additional data on *N*. *spinipes* available to date, including results obtained from this study [15, 48]. Embryonic development time and latency were found to not be normally distributed. Thus, the corresponding randomization functions were recalibrated from normal distributions to gamma distributions (as already had been done for naupliar and copepodite development). Due to its asymmetrical shape, the gamma distribution also accounts for ‘laggard’ animals which occur in real data sets [52–54]. The positive skewness of development time histograms is demonstrated exemplarily for the naupliar and copepodite development in Fig 2. Because not all previous studies differentiated between first, second or *n*th brood, just one parameter for brood size (mean with standard deviation) was calculated on all available brood size data at standard testing conditions. All individual level state variables and model parameters are presented in Table 1. On the population level, each life stage forms a distinct state variable comprising the sum of its individuals.

**Table 1:** State variables and parameters for simulation of individual copepods.

State variable	Description	Parameter	Value	Shape parameter alpha (α)^a^	CV	Unit
*N*_DEV_	Naupliar development	*pN*_DEV_	0.129	30.0	–	d^-1^
*C*_DEV_	Copepodite development	*pC*_DEV_	0.111	14.3	–	d^-1^
*L*	Latency	*pL*	0.308	26.1	–	d^-1^
*E*_DEV_	Embryo development	*pE*_DEV_	0.315	3.5	–	d^-1^
*BS*	Brood size	*pBS*	24.0	–	0.44	–
*NB*	Broods per female	*pNB*	3.64	–	0.37	–
*S*_NAU_	Naupliar survival	*pS*_NAU_	2.65 × 10^−3^	–	–	d^-1^
*S*_COP_	Copepodite survival	*pS*_COP_	4.83 × 10^−3^	–	–	d^-1^
*S*_ADU_	Adult survival	*pS*_ADU_	3.19 × 10^−3^	–	–	d^-1^

^a^Inverse-gamma distribution parameter.

CV = coefficient of variation; – = not applicable.

### Generation of new model functions

All regressions on experimental data were executed in SigmaPlot 13.0 (Systat Software, San Jose, CA). All model functions of temperature and food dependency are shown in Tables 2 and 3.

**Table 2:** Food dependence of daily ingestion rate per individual.

State variable	Description	Parameter	Function	R^2^	Unit
*IC*_NAU_	Naupliar ingested carbon	*pIC*_NAU_	*pIC*_NAU_ = 0.2981 × *C*_food_^2^ / (*C*_food_^2^ + 3.1993^2^)	0.98	μg C × d^-1^
*IC*_COP_	Copepodite ingested carbon	*pIC*_COP_	*pIC*_COP_ = 0.0677 × *C*_food_^2^ / (*C*_food_^2^ + 0.8491^2^)	0.95	μg C × d^-1^
*IC*_ADU_	Adult ingested carbon	*pIC*_ADU_	*pIC*_ADU_ = 0.2623 × *C*_food_^2^ / (*C*_food_^2^ + 1.5053^2^)	0.84	μg C × d^-1^

*C*_food_ = bottom food concentration (μg C × cm^-2^).

**Table 3:** Model functions of temperature and food dependence.

Parameter	Function type	Function	R^2^	Unit	Normalizing factor
Temperature dependence					
*pN*_DEV_	Exponential decay	*pN*_DEV_ = 1 / (5.3676 + 373.3086 × e^-0.3027 × t^)	1	d^-1^	5.85
	Bělehrádek normalized^a^	*pN*_DEV_ = 1 / (250 × (t + 7.2)^-1.86^ × 12.432)	–	d^-1^	5.85
*pC*_DEV_	Exponential decay	*pC*_DEV_ = 1 / (7.9317 + 43.4835 × e^-0.1545 × t^)	1	d^-1^	9.38
	Bělehrádek normalized^a^	*pC*_DEV_ = 1 / (250 × (t + 7.2)^-1.86^ × 19.956)	–	d^-1^	9.38
*pE*_DEV_	Exponential decay	*pE*_DEV_ = 1 / (3.13 + 70.612 × e^-0.2899 × t^)	1	d^-1^	3.25
	Bělehrádek normalized^a^	*pE*_DEV_ = 1 / (250 × (t + 7.2)^-1.86^ × 6.911)	–	d^-1^	3.25
*pL*	Bělehrádek normalized	*pL* = 1 / (250 × (t + 7.2)^-1.86^ × 6.860)	–	d^-1^	3.23
*BS*	Quadratic	*BS* = -0.5569 + 4.0229 × t - 0.143 × t^2^	1	–	
*pS*_NAU_	Quadratic	*pS*_NAU_ = (8.3922–0.8520 × t + 0.0216 × t^2^) / 100	1	d^-1^	975
*pS*_COP_	Quadratic	*pS*_COP_ = (3.7204–0.3801 × t + 0.0110 × t^2^) / 100	1	d^-1^	147
*pS*_ADU_	Quadratic	*pS*_ADU_ = (5.3501–0.5704 × t + 0.0157 × t^2^) / 100	1	d^-1^	250
Food dependence					
*pN*_DEV_	Power	*pN*_DEV_ = 0.0009 + 0.1789 × *IC*_NAU_^0.1943^	0.99	d^-1^	7.43
*pC*_DEV_	Power	*pC*_DEV_ = 0.0002 + 0.1827 × *IC*_COP_^0.2824^	0.99	d^-1^	11.8
*BS*	Exponential	*BS* = 33.2374 × (1 - e^-10.626 × *IC*ADU^)	0.90	–	0.0324
*pS*_NAU_	Rational	*pS*_NAU_ = 1 / (25.3937 + 2972.583 × *IC*_NAU_)	0.93	d^-1^	690
*pS*_COP_	Cubic	*pS*_COP_ = 0.0016–0.0175 × *IC*_COP_ + 5.4615 × *IC*_COP_^2^–73.4746 × *IC*_COP_^3^	0.56	d^-1^	333
*pS*_ADU_	Rational	*pS*_ADU_ = 1 / (12.8226 + 4504.1407 × *IC*_ADU_)	0.95	d^-1^	1111

^a^Optional model function presuming equiproportionality of embryonic and postembryonic stage development times at different temperatures.

– = not applicable.

Two different approaches were used to generate temperature functions for the duration related parameters *pN*_DEV_, *pC*_DEV_, and *pE*_DEV_. When evaluating the *Dc/Dn*-ratio for multiple calanoid, cyclopoid and harpacticoid copepod species, Hart did not find a significant link between the *Dc/Dn*-ratio and temperature [19]. These findings indicate equiproportionality [39, 55, 56] of developmental data and support the application of a single temperature function with fixed parameters to all stage durations. To generate such a function, Kulkarni et al. fitted Bělehrádek’s function [57] to six datasets from Hart’s compilation which they had normalized to a mutual testing temperature of 12°C [58]. In the first approach, we relatively adjusted this function to intersect with the 22°C-default duration values of *N*. *spinipes* (Table 1). In the second approach, a three-parameter exponential decay function was fitted independently to our measured data on naupliar, copepodite and embryonic development time, respectively, promising optimal curve fits. The corresponding inverse functions (since duration parameters in the model are reciprocal of actual durations) were implemented in the model as two optional functions. Since no temperature-related data on latency were available, only the Bělehrádek function was applied as described above. The parameters *BS*, *S*_NAU_, *S*_COP_ and *S*_ADU_ were expected to follow optimum functions. A second order polynomial (quadratic) function was used to generate convenient fits.

In order to describe food dependency dynamically and to allow for competitive interaction of copepods, life cycle parameters were related to the daily ingested carbon per animal in μg C instead of the actual food concentration. Considering the fact that *N*. *spinipes* is a bottom dwelling species, the test vessel bottom area was used to translate the provided food concentration to a bottom food concentration in μg C/cm^2^. Food ingestion rates at different bottom food concentrations were quantified fluorometrically. For nauplii, copepodites and adult animals (females), a Holling Type 3 functional response [59] with two parameters was fitted to the data (Table 2). While feeding intervals during the life cycle experiments varied between two and three days (during weekends), for simplification the feeding in model simulations takes place every second day. Due to consumption, the total amount of available food in the experiment continuously decreased within each feeding interval. To account for the food loss during simulations, ingested carbon of individuals is computed over 24 hours in discrete time steps of one minute. Every minute, the ingestion rate is recalculated for all living copepods within the treatment. The ingested carbon per minute is subtracted from the total food amount in the test vessel and added to the total carbon uptake of an individual for the current day. Currently, no reliable mathematical functions are known in which the copepod life cycle parameters addressed in this study can be directly linked to actual carbon intake without the demand for additional model variables. For that reason, model functions were simply created on best-fit approximations with commonly-used function types (Table 3).

For all food and temperature functions, normalizing factors were implemented. In this way, all functions can be applied relative to the default parameters which were calculated on multiple datasets.

While the source code of the original model was written in Delphi 2009 [60], it was in this study translated and modified in the programming language R [50]. The ‘shiny’ package [61] was used to build an interactive web application for model simulations. The source code can upon request be made available to interested parties by the corresponding author.

### Model verification

Simulations over 35 days were performed to reproduce the development test at all three temperature setups. The Bělehrádek function with shared coefficients supposes equiproportionality of the development times for different temperatures. However, results from this study did not support equiproportionality (see Results section). Therefore, the exponential decay functions were applied to all durational parameters except for latency, for which Bělehrádek’s function currently is the best available estimate. Development test setups at different food levels were simulated for a time span of 45 days. For all development test simulations, the initial number of nauplii was chosen in accordance with the precise number of nauplii at the start of the experiment. Reproduction of adult animals was disabled to simulate individual isolation. Temperature and food functions were directly applied without normalization to the default parameters (calculated on multiple datasets) to reproduce results from this study as accurately as possible. The Monte Carlo method was applied to calculate mean values and 95% confidence intervals of 1000 single simulations. Experimental data points were then plotted to visualize the accuracy of the model predictions.

The functionality of the reproduction submodel was assessed by repeatedly (*n* = 50) simulating individual fertilized females at different temperatures and food concentrations (corresponding to the experimental conditions) over a period of 15 days. Data of brood size and the brood-to-brood periods were compared to the theoretical values (directly derived from the model functions) in a one-sided t-test (α = 5%).

## Results

### Life cycle tests

The measured minimum, maximum, and mean temperature over the test period are presented in Table 4 for each temperature setup. Furthermore, the mean daily minimum and maximum with corresponding standard deviations are presented as a measure of variability. Laggard animals were found in all test setups, resulting in a positive skewness in the frequency distribution of development times. All development time data failed in either one or both of the pretests for normality and equal variance. The same was true for the measured clutch sizes per brood (brood sizes). Despite these slight deviations from normality, we still regard mean and standard deviation as convenient measures for a tabular presentation of the data (Table 5). *Dc/Dn*-ratios are presented as a measure of temperature-dependent proportionality of postembryonic development times. The naupliar and copepodite mortality and sex ratio data of all treatments are shown in Table 6. The ‘no-food’ treatment was excluded from Tables 5 and 6 since no individual in this treatment survived long enough to reach even the first copepodite stage. Even though the number of broods per female was not assessed quantitatively as an endpoint in the present study, it was noted that much fewer females developed successful broods at the highest temperature setup and at the lowest food concentrations.

**Table 4:** Measured temperatures throughout the temperature experiments.

Temperature Setup	Mean of daily means [°C]	Total minimum [°C]	Total maximum [°C]	Mean ± SD of daily minima [°C]	Mean ± SD of daily maxima [°C]
15°C	14.86	13.6	17.3	14.16 ± 0.28	15.56 ± 0.82
20°C	19.34	18.3	20.2	18.78 ± 0.14	19.90 ± 0.21
25°C	24.90	24.0	25.2	24.61 ± 0.24	25.19 ± 0.06

SD = standard deviation.

**Table 5:** Embryonic, naupliar and copepodite development times, *Dc/Dn*-ratios and brood sizes at all temperature and food setups.

Setup	Naupliar development [d]	Copepodite development [d]	*Dc/Dn*	Embryo development [d]	Brood size
**Temperature setups**					
**15°C**	9.52 ± 2.19 (61)	12.31 ± 2.04 (55)	1.29	4.08 ± 0.36 (37)	27.65 ± 6.59 (34)
**20°C**	6.44 ± 0.66 (66)	10.12 ± 2.89 (65)	1.57	3.39 ± 0.76 (136)	23.76 ± 10.78 (139)
**25°C**	5.57 ± 1.18 (60)	8.86 ± 1.29 (57)	1.59	3.18 ± 0.87 (11)	10.95 ± 4.94 (43)
**Food setups**					
**1.25 × 10**^**4**^ **cells/mL**	16.10 ± 3.73 (20)	20.50 ± 6.81 (4)	1.27	–	–
**2.5 × 10**^**4**^ **cells/mL**	12.86 ± 1.80 (28)	17.52 ± 9.64 (21)	1.36	*ND*	12 ± 2.55 (5)
**5 × 10**^**4**^ **cells/mL**	11.74 ± 2.69 (31)	13.32 ± 4.36 (28)	1.13	*ND*	20.50 ± 10.77 (40)
**1.25 × 10**^**5**^ **cells/mL**	8.51 ± 1.29 (34)	12.55 ± 4.85 (32)	1.47	*ND*	25.88 ± 9.26 (51)
**2.5 × 10**^**5**^ **cells/mL**	7.66 ± 1.44 (34)	11.50 ± 3.32 (31)	1.50	*ND*	30.50 ± 9.91 (52)
**5 × 10**^**5**^ **cells/mL**	6.71 ± 0.50 (35)	11.21 ± 2.37 (34)	1.67	*ND*	35.35 ± 7.59 (49)

Measured data are presented as mean ± standard deviation (n-value in parenthesis). *Dc/Dn* = copepodite to naupliar duration ratio; – = not applicable; *ND* = not determined.

**Table 6:** Stage mortality and sex ratio data at all temperature and food setups.

Setup	Naupliar survival		Copepodite survival		Sex ratio	
	Alive	Dead	Alive	Dead	Female	Male
**Temperature setups**						
**15°C**	61	5	55	6	22	31
**20°C**	66	3	65	0	39	24
**25°C**	60	9	57	3	40	10
**Food setups**						
1.25 * 10^**4**^ **cells/mL**	19	17	10	9	0	3
2.5 * 10^**4**^ **cells/mL**	28	8	24	4	4	17
5 * 10^**4**^ **cells/mL**	34	2	32	2	3	25
1.25 * 10^**5**^ **cells/mL**	35	1	33	2	10	21
2.5 * 10^**5**^ **cells/mL**	34	2	32	2	8	23
5* 10^**5**^ **cells/mL**	35	1	35	0	11	23

When applying the Kruskal-Wallis test with Bonferroni correction to metric data from the temperature experiment, a significant (*p* ≤ 0.05) difference among the treatments was found in naupliar development (*p* = 5.7 × 10^−29^), copepodite development (*p* = 3.4 × 10^−13^), embryonic development (*p* = 6.2 × 10^−10^) and brood size (*p* = 3.5 × 10^−18^). Furthermore, naupliar development (*p* = 2.7 × 10^−29^) and brood size (*p* = 1.4 × 10^−11^) differed significantly among the food treatments. The copepodite development appeared to also increase with decreasing food concentrations. However, this trend was not significant (*p* = 0.12). Altogether, the measured data indicate that temperature was inversely correlated to development times as well as to brood size. The same was true for the food concentration, except for the brood size, which increased at greater food concentrations. *Dc/Dn*-ratios increased by 23% between the 15°C setup and the 25°C setup. Also, when comparing *Dc/Dn*-ratios for increasing food concentrations, a steady increase (31% from the lowest to the highest) could be observed. The 5 × 10^4^ cells/mL treatment with the altogether lowest *Dc/Dn*-ratio forms the only exception to this general trend.

According to Fisher’s exact test, the naupliar mortality did not differ significantly (*p* ≤ 0.05) among the temperature treatments (*p* = 0.17). For the copepodite mortality, however, a significant difference was found (*p* = 0.037). The data indicate a temperature optimum curve for survival with the optimum located closely to 20°C. Mortality appeared to be inversely correlated to food concentration. Fisher’s exact test revealed a significant difference for both naupliar (*p* = 9.8 × 10^−9^) and copepodite mortality (*p* = 1.5 × 10^−6^). The sex ratio shifted significantly toward more females per males (from 0.7 to 4.0) with increasing temperatures from 15 to 25°C (*p* = 3.3 × 10^−4^). No significant link between the sex ratio and food concentration was found (*p* = 0.29).

### Simulations

**Fig 3:**
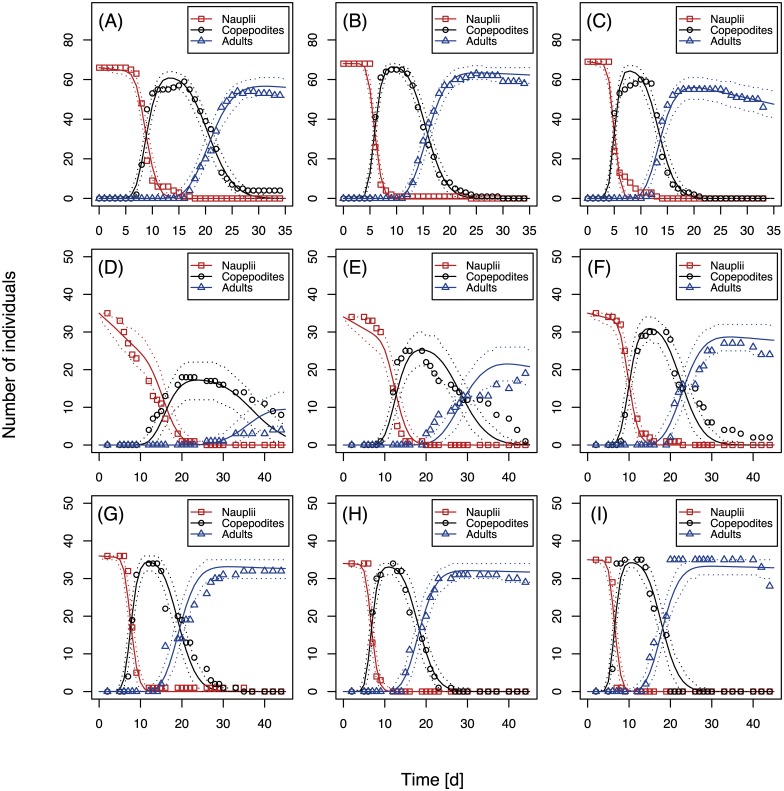
Simulations of the development test for all temperature and food setups. Abundance over time is shown for nauplii, copepodites, and adult animals at three different testing temperatures: 15 (A), 20 (B) and 25°C (C) with a standard food concentration of 2.5 × 10^5^ algal cells/mL and six different food concentrations: 0.125 (D), 0.25 (E), 0.5 (F), 1.25 (G), 2.5 (H) and 5 (I) × 10^5^ algal cells/mL at a standard temperature of 22°C. Solid lines represent mean values of 1000 single simulations. Dotted lines indicate 95% confidence intervals. Experimental data are displayed as empty shapes.

Model simulations reproducing the development test at all experimental temperature and food setups are shown in Fig 3. Model simulations generally matched the experimental data very well. Only a few experimental data points were located outside the 95% confidence interval of the simulations.

In model simulations of individual females, mean brood sizes and brood-to-brood periods corresponded closely to the expected values (Table 7). No significant difference (*p* ≤ 0.05) was found.

**Table 7:** Simulated brood-to-brood periods and brood sizes at all testing conditions.

Setup	Brood-to-brood period		Brood size	
	Simulation	Theoretical value	Simulation	Theoretical value
**Temperature setups**				
**15°C**	3.96 ± 0.79 (50)	4.04	29.30 ± 13.74 (50)	27.64
**20°C**	3.33 ± 0.72 (50)	3.39	26.10 ± 10.97 (50)	23.82
**25°C**	3.21 ± 0.58 (50)	3.18	10.74 ± 5.16 (50)	10.70
**Food setups**				
**2.5 × 10**^**4**^ **cells/mL**	*ND*	*ND*	11.26 ± 3.57 (50)	11.13
**5 × 10**^**4**^ **cells/mL**	*ND*	*ND*	21.48 ± 10.31 (50)	22.51
**1.25 × 10**^**5**^ **cells/mL**	*ND*	*ND*	31.00 ± 12.27 (50)	29.49
**2.5 × 10**^**5**^ **cells/mL**	*ND*	*ND*	31.32 ± 12.99 (50)	30.58
**5 × 10**^**5**^ **cells/mL**	*ND*	*ND*	26.92 ± 15.75 (50)	31.11

Simulated data are presented as mean ± standard deviation (n-value in parenthesis). Mean theoretical values were directly derived from the corresponding model functions. *ND* = not determined.

## Discussion

### Embryonic and postembryonic development

Based on earlier studies, we considered the 20°C temperature setup (with 2.5 × 10^5^ algal cells/mL) and the two highest food setups (2.5 and 5.0 × 10^5^ algal cells/mL at 22°C) to represent close to optimal conditions [11, 13, 15]. In these experimental setups we measured mean naupliar development times of 6.44 ± 0.66, 7.66 ± 1.44 and 6.71 ± 1.29, respectively, which are in line with literature data for this species [11, 13, 15, 51]. Copepodite development times at the same conditions were about 50% higher than naupliar development times (10.12 ± 2.89, 11.50 ± 0.66 and 11.50 ± 0.66, respectively). Literature values for the copepodite development time of *N*. *spinipes* under control conditions show high variations but are generally similar to the naupliar development time. One reason for the high variability in literature data and the relatively high values measured in this study may be the use of different approaches to confirm the adult stage of *N*. *spinipes*. The fifth copepodite stage is easy to be confused with the adult stage. We consider the method used in this study (i.e. counting exoskeletons for each molt) as the most reliable method. The mean embryonic development time at the 20°C temperature setup (3.39 ± 0.76) is, again, consistent with data available in literature [11]. For all embryonic and postembryonic development data, the duration of development decreased significantly with increasing temperatures. Furthermore, postembryonic development times were found to increase with decreasing food concentrations. This trend was found to be significant for naupliar but not for copepodite development time which showed much higher variability at low food concentrations. In general, these findings are consistent with numerous copepod temperature and food studies presented in the past [20, 38, 41, 42, 62–68]. In a synopsis of copepod postembryonic development data, Hart found no temperature dependence of the copepodite to naupliar duration ratio *Dc/Dn*, indicating an equiproportional reduction of naupliar and copepodite stage durations with increasing temperatures [19]. Such equiproportionality is a prerequisite for the use of generalized temperature equations for different stage durations as done by Kulkarni et al. [58]. In the present study, the *Dc/Dn*-ratio increased by 23% from 15°C to 25°C. Although this shift seems rather distinct, similar fluctuations without distinguishable trends can be found in Hart’s collation of data. At this point, no definite conclusion can be drawn about the existence or nonexistence of equiproportionality of harpacticoid copepod stage durations. As already stated by Sarvala in 1979 [62], not all data support equiproportionality, but it may certainly be used as a first approximation.

Moreover, the *Dc/Dn*–ratio in this study was found to be correlated to the food concentration. In contrast, Hart’s data revealed an inverse correlation trend which was especially distinct for freshwater copepods. However, the pattern was not as clear for marine species whose *Dc/Dn*-ratio often showed only slight irregular fluctuations. Taken as a whole, the findings of this study point toward a higher developmental sensitivity of nauplii toward alterations in temperature and food concentration when compared to copepodites.

### Mortality

Only a few animals (4%) died before reaching the adult stage at the 20°C setup. For 15 and 25°C, overall mortality was somewhat higher (17% in both setups). Thus, we assume an optimum temperature for survival close to 20°C. Although *N*. *spinipes* naturally inhabits colder environments, it is likely that the tested strain of *N*. *spinipes* has adapted to the laboratory conditions (22°C) on a genetic level after 40 years of continuous culture. Even though cultures were acclimatized for at least 18 days at 15 and 25°C, only non-genetic adaptation may have occurred at these temperature setups. Non-genetic adaptation has earlier been found to be related to alterations in lethal limits, activity, metabolism, reproduction and other physiological processes and may, therefore, be the cause of the increase in mortality [69]. In accordance with literature [41, 44], mortality of *N*. *spinipes* was generally enhanced with decreasing food concentrations.

### Sex ratio

Sex ratios at the three temperature setups differed significantly from each other. Regarding the increasing ratio of females per male from 0.7 to 4.0 with increasing temperatures, a true shift seems rather obvious. However, relatively high variations in the sex ratio of equally treated control setups have previously been reported [45]. The mortality of 17% for the total development period from nauplius to adult at the 25°C setup may be one cause for the sex ratio shift. However, even if it were assumed that only male animals died before reaching sexual maturity, this cannot fully explain the female prevalence. If sex change is accepted as a possible driver of sex ratio shifts [70, 71], observations of adult sexes may not even be representative of a fixed underlying sex ratio at birth. No significant relationship between the sex ratio and food concentration was found in this study. However, a trend toward more males per female was observed in all treatments which is rather unusual for harpacticoid copepods [72]. More research on sex ratios seems indispensable since the density of males is of great relevance for the reproductive output of a population [70, 73].

### Reproduction

Beside embryonic development time, the brood size per clutch was also found to decrease with increasing temperatures (from 27.65 ± 6.59 at the 15°C setup to 10.95 ± 4.94 at the 25°C setup). Similar trends have been observed for other copepod species [63, 74, 75]. At high temperatures, the metabolic costs per animal rise more rapidly than does assimilation. If the food passage through the gut is concurrently accelerated, the efficiency of digestion inevitably decreases at high temperatures [62]. Higher metabolic costs for development and survival may lead to lower energetic investment in broods and cause a decreased number of offspring per brood. The adverse effect on reproduction caused by reduced brood size at the 25°C temperature setup strongly overweighed the promotive effect of reduced embryonic development time observed in this study. Brood size was also found to be inversely correlated to the food concentration. This, again, reflects literature results and can be simply explained by the lack of energy that comes with food deprivation [49, 67, 75, 76]. Despite not having been assessed quantitatively in the design of this study, much fewer females were found to developed successful broods at the highest temperature setup and at the lowest food concentrations. Taking into account the number of nauplii per brood and the number of broods per female, we conclude that high temperatures and low food concentrations strongly affect the reproductive output of *N*. *spinipes* in a negative way.

### Model functions and applicability

As stated before, Bělehrádek’s function with fixed coefficients can only be properly applied for different stage development times (embryonic, naupliar and copepodite development) if equiproportionality among stage durations exists. Due to the apparent lack of equiproportionality in the development data of this study, all model verification simulations were performed with the exponential decay development functions. The functions had been fitted independently for embryonic, naupliar and copepodite development. In contrast to Bělehrádek’s function, the use of exponential one phase decay as a temperature-development function was not based on scientific literature but was chosen as a promising best possible fit function. Nonetheless, it has to be noted that many of the most popular temperature functions have emerged from creating optimal fit curves for existing data rather than from a theoretical and mechanistic basis [38]. In that respect, the exponential one phase decay function proved very suitable for the present data.

Overall, the verification simulations convincingly reproduced the experimental data obtained in this study. In simulations reproducing the development test, however, several experimental data points were not covered by the 95% confidence intervals. This can primarily be explained by the variability in individual development times which were modeled my means of a gamma distribution around the mean. While the corresponding gamma distribution shape parameters were calibrated on control data from multiple studies [11, 13, 15, 48, 51], the distribution shapes differed slightly in the temperature and food experiments of this study. These deviations were not accounted for in the model simulations (to avoid overfitting) and thus, data variability was not always covered perfectly.

In this study, all life cycle experiments were executed at optimal conditions for either food or temperature while no experiments were performed in which both factors deviated from their optimum at the same time. However, lifespan and reproduction data on the harpacticoid copepod *Tisbe battagliai* presented by Williams and Jones [75] indicate that temperature and food quantity have interactive effects. More experiments at suboptimal environmental conditions in combination with toxic exposure need to be performed to assess such interactive effects in *N*. *spinipes*. In the context of global climate change, we strongly support the consideration of combined environmental stress scenarios in environmental risk assessment of chemicals. When taking into account toxic exposure scenarios, and additional climate change-related stressors (e.g. salinity, pH, hypoxia and ultraviolet radiation), the simultaneous use of multiple stress functions is indispensable for informative model predictions [34, 77]. Multivariate stress equations may be established to yield more precise model predictions on interactive stress effects [19]. Since there is a demand for more effect-based monitoring tools in the EU water framework directive, we furthermore advocate combining *in vivo* bioassays and population modeling as a cost effective higher tier approach to assess population-level risks [78].

## Conclusions

Qualitatively, the measured effects of temperature and food availability on the life cycle parameters of *N*. *spinipes* are consistent with previous studies on related species. High temperatures and low food concentrations were found to diminish the reproductive output of *N*. *spinipes* and thus are likely to have negative implications on the population level. The individual based copepod model was found to reliably reproduce the experimental data of development and reproduction, obtained in the laboratory. Further research needs to be conducted on the combined effects of climate change-related and chemical stressors in *N*. *spinipes* to allow for accurate model predictions of realistic exposure conditions in the field.
